# Mutants of GABA Transaminase (POP2) Suppress the Severe Phenotype of *succinic semialdehyde dehydrogenase* (*ssadh*) Mutants in Arabidopsis

**DOI:** 10.1371/journal.pone.0003383

**Published:** 2008-10-10

**Authors:** Frank Ludewig, Anke Hüser, Hillel Fromm, Linda Beauclair, Nicolas Bouché

**Affiliations:** 1 Botanical Institute, University of Cologne, Cologne, Germany; 2 Department of Plant Sciences, Faculty of Life Sciences, Tel Aviv University, Tel Aviv, Israel; 3 INRA, Institut Jean Pierre Bourgin, Station de Génétique et d'Amélioration des Plantes, UR254, Versailles, France; Umeå Plant Science Centre, Sweden

## Abstract

**Background:**

The γ-aminubutyrate (GABA) shunt bypasses two steps of the tricarboxylic acid cycle, and is present in both prokaryotes and eukaryotes. In plants, the pathway is composed of the calcium/calmodulin-regulated cytosolic enzyme glutamate decarboxylase (GAD), the mitochondrial enzymes GABA transaminase (GABA-T; POP2) and succinic semialdehyde dehydrogenase (SSADH). We have previously shown that compromising the function of the GABA-shunt, by disrupting the *SSADH* gene of Arabidopsis, causes enhanced accumulation of reactive oxygen intermediates (ROIs) and cell death in response to light and heat stress. However, to date, genetic investigations of the relationships between enzymes of the GABA shunt have not been reported.

**Principal Findings:**

To elucidate the role of succinic semialdehyde (SSA), γ-hydroxybutyrate (GHB) and GABA in the accumulation of ROIs, we combined two genetic approaches to suppress the severe phenotype of *ssadh* mutants. Analysis of double *pop2 ssadh* mutants revealed that *pop2* is epistatic to *ssadh*. Moreover, we isolated EMS-generated mutants suppressing the phenotype of *ssadh* revealing two new *pop2* alleles. By measuring thermoluminescence at high temperature, the peroxide contents of *ssadh* and *pop2* mutants were evaluated, showing that only *ssadh* plants accumulate peroxides. In addition, *pop2 ssadh* seedlings are more sensitive to exogenous SSA or GHB relative to wild type, because GHB and/or SSA accumulate in these plants.

**Significance:**

We conclude that the lack of supply of succinate and NADH to the TCA cycle is not responsible for the oxidative stress and growth retardations of *ssadh* mutants. Rather, we suggest that the accumulation of SSA, GHB, or both, produced downstream of the GABA-T transamination step, is toxic to the plants, resulting in high ROI levels and impaired development.

## Introduction

γ-aminobutyric acid (GABA) is a non-protein amino acid predominantly associated with neurotransmission in the mammalian brain but also found in non-neuronal cells [1], in plants [2], in unicellular eukaryotes [3], and in prokaryotes [4]. The production of GABA in plants is significantly enhanced in response to various biotic and abiotic stresses [2], [5]–[7]. Recent studies suggest it has a major role in plant primary metabolism [8], [9] but other functions of this metabolite remain unknown.

**Figure 1:**
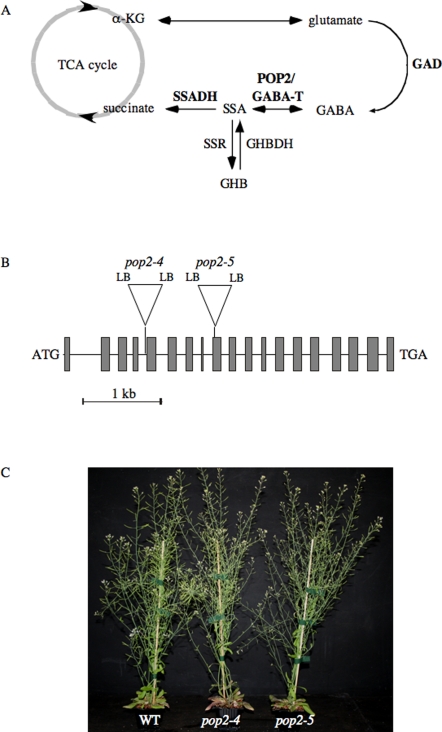
The GABA shunt metabolic pathway and the *pop2* mutants. (A) Schematic presentation of the GABA shunt metabolic pathway. The GABA shunt is composed of three enzymes (in bold). The cytosolic glutamate decarboxylase (GAD) catalyses the irreversible decarboxylation of glutamate to produce GABA. GABA is transported in the mitochondria to be converted into succinic semialdehyde (SSA) by a GABA transaminase (GABA-T/POP2). SSA is then oxidized by a succinic semialdehyde dehydrogenase (SSADH) to form succinate, which enters the tricarboxylic acid (TCA) cycle. In animals and possibly in plants, SSA can be converted into γ-hydroxybutyric acid (GHB) by a SSA reductase (SSR) and GHB into SSA via another enzyme, the GHB dehydrogenase (GHBDH). α-KG; α-ketoglutarate. (B) Characterization of *pop2-4* and *pop2-5* T-DNA mutants. Gene structure of the *GABA-T*/*POP2* (At3g22200) ORF and T-DNA insertions in both *pop2-4* (SAIL_1230_C03) and *pop2-5* (GABI_157D10) mutants. Exons are represented by boxes (drawn to scale). *LB* designs the left T-DNA borders. Junctions between the T-DNAs and the gene were sequenced. (C) Phenotype of the *pop2-4* and *pop2-5* mutants compared to WT (Col) plants. Seeds were sown on soil and grown for a total of 45 days in the greenhouse before being photographed.

GABA synthesis (Figure 1A) from glutamate is controlled by the cytosolic glutamate decarboxylase (GAD), a Ca^2+^/calmodulin regulated enzyme in plants [10]. GABA is catabolized in mitochondria through the GABA-shunt [2], a metabolic pathway that bypasses two successive steps of the tricarboxylic acid cycle catalyzed by α-ketoglutarate dehydrogenase and succinyl CoA ligase. The enzymes involved in GABA catabolism are GABA transaminase (GABA-T) which converts GABA to succinic semialdehyde (SSA), and succinic semialdehyde dehydrogenase (SSADH) which oxidizes SSA to succinate coupled to NADH production. Hence, GABA is a metabolite *en route* from glutamate to the tricarboxylic acid cycle which provides succinate and NADH to the respiratory machinery. The production of succinate via the GABA-shunt seems to be of primary importance when the TCA cycle does not provide enough succinate. In fact, transgenic plants exhibiting decreased expression of the succinyl CoA ligase present a mild phenotype and slightly reduced rates of respiration; in these plants, GAD activities are increased and the production rate of succinate derived from the GABA-shunt is elevated, thus compensating the deficiency of succinate production by the TCA cycle [9]. SSA can also be converted into γ-hydroxybutyric acid (GHB) by a succinic semialdehyde reductase present in animals and recently discovered in plants [11].

In humans, SSADH deficiency, known as GHB aciduria, is a genetically inherited disease causing non-specific neurological disorders due to the accumulation of GHB and GABA in the brain [12]. GHB was shown to accumulate in mice deficient for SSADH [13] as well as in Arabidopsis *ssadh* knockout mutants [14]. GABA and GHB are playing crucial roles as neurotransmitters by binding to receptors in the mammalian brain. In plants, evidence is lacking to assign a function as signaling molecules to them because plant receptors binding GABA or GHB are still to be identified. Genes homologous to ionotropic glutamate receptors of animals are present in the genome of Arabidopsis [15], [16] and encode proteins that likely mediate sodium or calcium entry into cells [17]. Very recently, the characterization of a short-root mutant of *Oryza sativa* that has defects in a glutamate receptor gene (*GLR3.1*) provided genetic and molecular evidence for an important function of *GLR3.1* in seedling development [18]. Whether GABA could possibly interact with glutamate receptors in plants remains an open question [19]. Another putative GABA receptor could be the high affinity GABA transporter (AtGAT1) localized in the plasma membrane of Arabidopsis and expressed under conditions of elevated GABA content like mechanical wounding or senescence [20].

A GABA concentration gradient is essential for the growth and guidance of pollen tubes and suggests that this amino acid is involved in intercellular signaling [21]. The *pop2* (*pollen-pistil incompatibility2*) mutant is unable to produce a functional GABA-T enzyme, and, as a consequence, this leads to growth inhibition and misguidance of *pop2* pollen tubes in *pop2* pistils because the GABA gradient guiding pollen tubes is disturbed. GABA binding sites (i.e. putative receptors) were detected on protoplast membranes of both pollen and somatic cells using a fluorescent probe [22]. The main question raised by these studies is whether GABA itself serves as a signaling molecule in plants. Recent findings point toward a role of GABA as a signal between plants and pathogenic bacteria since GABA can modulate quorum sensing in *Agrobacterium tumefaciens* [23], [24]. A role for GABA in mediating responses to volatiles produced during wounding or pathogen attacks was proposed since some of these volatiles induce GABA accumulation, and *pop2* alleles (*her1*) were isolated in mutagenesis screens to select Arabidopsis mutants with altered responses to such volatiles [25].

We have previously shown that compromising the function of the GABA-shunt causes enhanced accumulation of reactive oxygen intermediates (ROIs) and cell death in response to light and heat stress [26]. We described the phenotype of *ssadh* mutants impaired in the last step of the GABA-shunt. In brief, when grown under standard light conditions, the four isolated *ssadh* homozygous mutants (*ssadh-1* to *ssadh-4*) are dwarfs and present necrotic lesions and bleached spots on leaves, reduced leaf area, lower chlorophyll content, and fewer flowers compared to wild type plants. In addition, *ssadh* mutants are more sensitive to at least two types of environmental stresses. The development of *ssadh* mutants exposed to UV-B light or heat stress is significantly retarded and associated with the appearance of necrotic lesions. H_2_O_2_ contents are increased in *ssadh* plants exposed to stress, as shown by 3,3-diaminobenzidine (DAB) staining and direct H_2_O_2_ quantification [26]. Thus, the phenotype of *ssadh* plants grown in standard conditions is probably associated with the UV-B light contained in white light. To explain the accumulation of H_2_O_2_ in *ssadh* mutants and its severe growth retardation, we mentioned two possible hypotheses [26]. First, the lack of supply of essential metabolites to the TCA cycle (particularly succinate and NADH), and secondly, the accumulation of a toxic compound (SSA or GHB, or both) due to the block in the metabolism of SSA. Here we combined two genetic approaches to suppress the severe phenotype of *ssadh* mutants, with physiological and metabolic investigations of single mutants, double mutants, and second-site suppressors to elucidate the cause of the *ssadh* phenotype and the role of SSA, GABA and GHB in the accumulation of ROIs.

## Results

### *pop2* is epistatic to *ssadh*

To dissect the phenotypic effects of *ssadh* mutations, we first crossed *ssadh* mutants with *pop2* mutants. Such *pop2 ssadh* double mutants are expected to lack the potential toxic compounds SSA, GHB (Figure 1A), or metabolites derived from their catabolism. However, *pop2 ssadh* double mutants would not be able to supply NADH and succinate to the TCA cycle via the GABA shunt, similar to *ssadh* mutants. Several mutations in the *GABA-T* (*POP2*) gene encoding the enzyme degrading GABA into SSA (Figure 1A), were described (*pop2-1*, *pop2-2* and *pop2-3* mutants; [21]). By comparing the *GABA-T* (At3g22200) genomic sequence of Arabidopsis with T-DNA flanking genomic sequences deposited in the databases we identified two additional *pop2* alleles designated *pop2-4* and *pop2-5* (Figure 1B). The genomic DNAs of the mutants were characterized by PCR (Materials and Methods) and the junctions between the T-DNAs and the gene were sequenced. Plants homozygous for the *pop2-4* or *pop2-5* mutations were phenotypically similar to other *pop2* alleles [21]. Homozygous plants grew as wild type but were partially sterile with no or very small siliques (Figure 1C).

**Figure 2:**
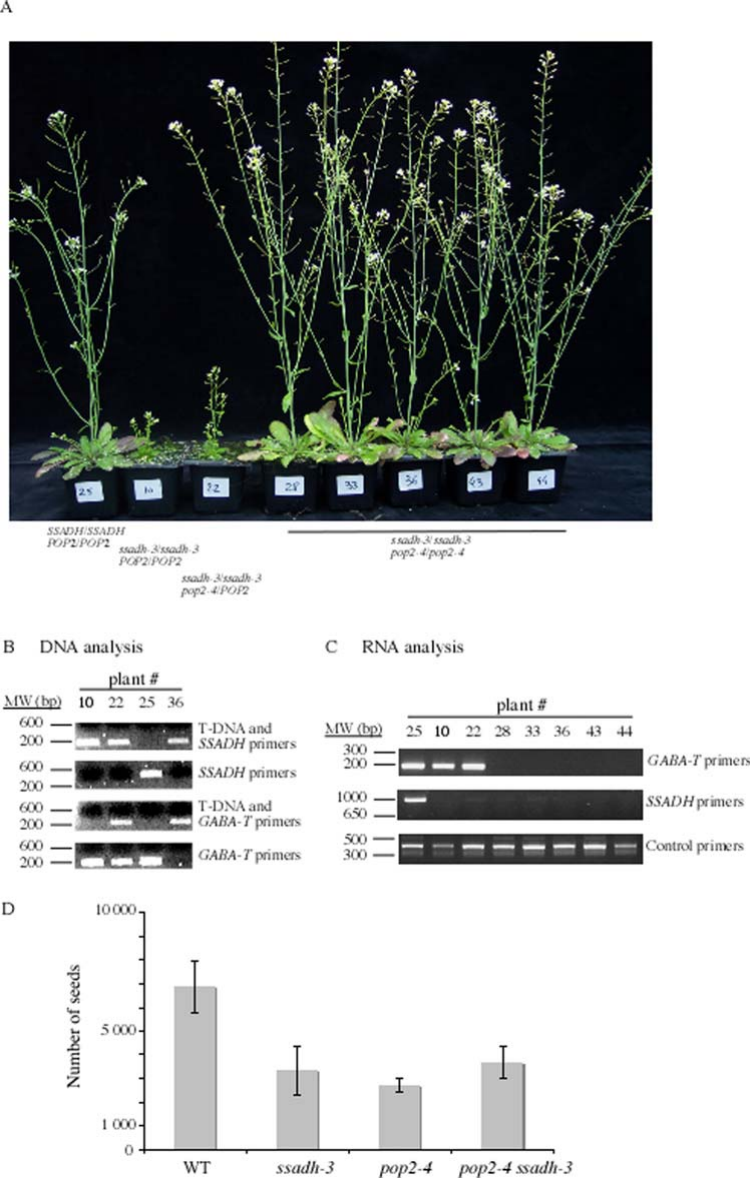
*pop2-4* is epistatic to *ssadh-3*. (A) Phenotype of six weeks old F2 plants resulting from a cross between *pop2-4* and *ssadh-3* homozygous plants. Genotypes are indicated, namely wild type (plant #25), *ssadh-3*/*ssadh-3* (plant #10), *ssadh-3*/*ssadh-3 pop2-4*/*POP2* (plant #22) and *ssadh-3*/*ssadh-3 pop2-4*/*pop2-4* double mutants (plants #28 to 44). (B) PCR analysis of F2 plants shown in (A). DNAs from F2 plants were subjected to a PCR amplification using *SSADH* or *GABA-T*/*POP2* gene specific oligos (Materials and Methods) and the T-DNA left border oligo corresponding to SAIL lines (LB3). MW; DNA molecular weight. (C) Expression analysis by RT-PCR of *GABA-T/POP2* and *SSADH* mRNAs in F2 plants shown in (A). Total RNAs from F2 plants were extracted and used as templates for reverse transcription (Materials and Methods). (D) Average number of seeds for WT (Col-0), *pop2-4*, *ssadh-3* and the corresponding double mutants. Seeds were counted for a total of 4 plants per genotype.

**Figure 3:**
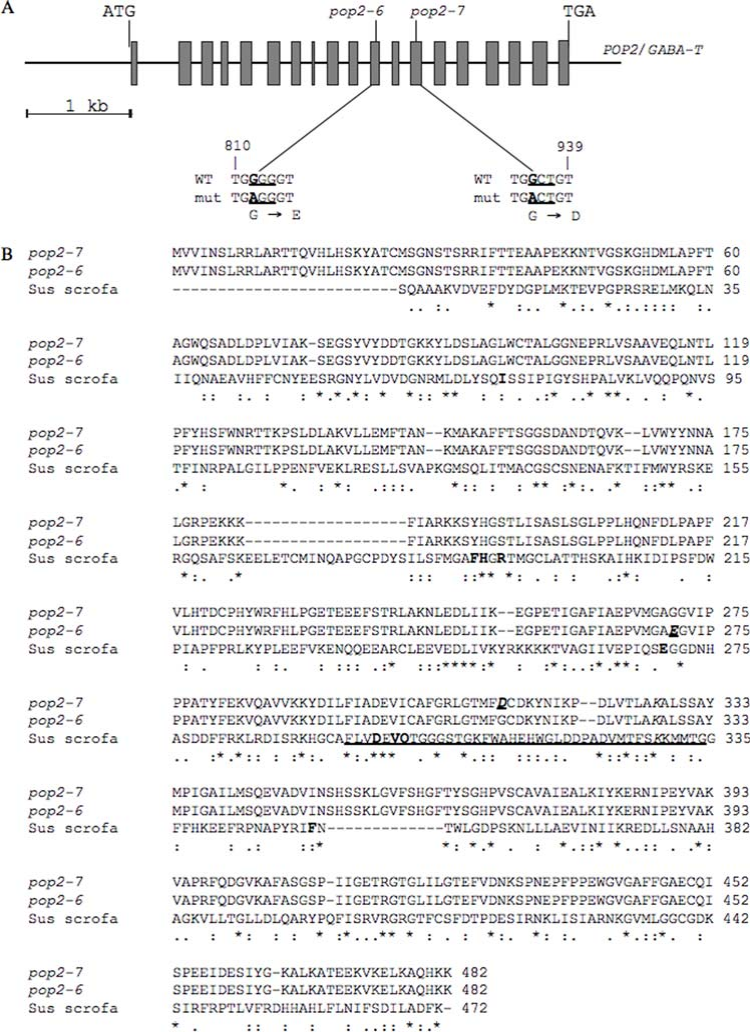
GABA-T nucleotide and amino acid sequences of *pop2-6* and *pop2-7* EMS mutants. (A) Position and nature of the point mutations (in bold) in the new *pop2 alleles*. Both *pop2-6* and *pop2-7* have a G→A transition resulting in the amino acid substitution G271E and G312D, respectively. The codons that have been changed in the mutants are underlined. (B) Comparison of the GABA-T amino acid sequences of Arabidopsis *pop2-6* and *pop2-7* and of *Sus scrofa* (GenBank accession 1OHV_A). ‘*’ indicates residues that are identical, ‘:’ homologous and ‘.’ similar . The active site residues of the pig enzyme are in bold, the pyridoxal phosphate recognition site is underlined, the attachment site is in italics, and the mutated residues are underlined, in bold and italics.

We crossed plants homozygous for the *ssadh-3* allele with heterozygous POP2/*pop2-4* plants. The resulting F1 and F2 plants were genotyped by PCR using oligos specific for *pop2-4* or *ssadh-3* T-DNA inserts (Figure 2B). F2 plants carrying only the *ssadh-3* mutation in the homozygous state exhibited a phenotype reminiscent of *ssadh* mutants (Figure 2A; plants #10 and #22) while plants homozygous for the *pop2-4* mutation exhibited a phenotype reminiscent of *pop2* mutants (data not shown). Double *pop2-4 ssadh-3* mutants homozygous for both mutations displayed a *pop2* phenotype. Indeed, *pop2 ssadh* double mutants grew as wild type plants but failed to form mature siliques (Figure 2A; plants #28 to 44) and produced less seeds (Figure 2D). The presence of the *SSADH* and *GABA-T* mRNAs was assessed in the F2 plants, by reverse transcription and PCR amplification using primers specific for *SSADH* or *GABA-T* cDNAs. The full-length *GABA-T*/*POP2* mRNA could not be detected in total RNA extracted from plants homozygous for the *pop2-4* mutation (Figure 2C; *GABA-T primers*; plants #28 to 44) in contrast to *POP2*/*POP2* (Figure 2C; *GABA-T primers*; plants #25 and 10) or *pop2-4*/POP2 (Figure 2C; *GABA-T primers*; plant #22) siblings. Similarly, the full-length *SSADH* mRNA could not be detected in total RNA extracted from plants homozygous for *ssadh-3* (Figure 2C; *SSADH primers*; plants #10 to 44) in contrast to wild type siblings (Figure 3C; *SSADH primers*; plant #25). Amplification of an elongation factor mRNA worked equally well on all RNA templates (Figure 2C; *control primers*). Thus, full length mRNAs corresponding to *SSADH* or *GABA-T* could not be detected in the double *pop2-4 ssadh-3* mutant. We confirmed these results by crossing other *pop2* and *ssadh* mutant alleles. Heterozygous *ssadh-2* mutants as pollinators were crossed to *pop2-5* plants, and the resulting F1 and F2 plants were genotyped by PCR using oligos specific for the *pop2-5* or *ssadh-2* T-DNA inserts (Materials and Methods). Double *pop2-5 ssadh-2* mutants displayed a phenotype characteristic of *pop2* mutants (Figure S1). Altogether, these results indicate that *pop2* is epistatic to *ssadh* because both *pop2-4 ssadh-3* and *pop2-5 ssadh-2* double mutants are similar to *pop2* mutants.

### EMS-induced mutations in the *POP2* gene suppress the phenotype of the *ssadh* mutation

To gain broader insight into the GABA metabolic pathway and potentially also into regulators of the pathway in Arabidopsis, we decided to isolate suppressors of the *ssadh* mutation. About 30,000 *ssadh-2* seeds were mutagenized with ethyl methanesulfonate (EMS; Materials and Methods). None of the resulting M1 plants grew significantly better than *ssadh-2* plants, suggesting that no dominant EMS-induced mutations could rescue *ssadh-2*. Out of 60,000 M2 plants, 137 with improved growth were isolated, potentially carrying recessive suppressor mutations rescuing the *ssadh* phenotype. The *GABA-T* gene was sequenced from ten plants that phenotypically resembled *pop2* mutants, and two new *pop2* mutants with single point mutations were identified designated *pop2-6* and *pop2-7* (Figure 3A). In *ssadh-2 pop2-6*, a nucleotide exchange from G to A occurred in exon 11 at position 812 of the *GABA-T* coding sequence, resulting in an amino acid substitution from glycine to glutamate (Figure 3A) adjacent to an active site residue (Figure 3B; [27]). This line was lost due to its sterility. Plants of another isolated suppressor mutant (*ssadh-2 pop2-7*) were found to have a nucleotide transition from G to A in exon 13 at position 935 of the *GABA-T* coding sequence, causing the replacement of glycine with aspartate (Figure 3A) located in the recognition site for binding of the cofactor pyridoxal phosphate (Figure 3B; [27]). Altogether, our data show that mutations in the *GABA-T* gene can suppress the *ssadh* phenotype and that *pop2* mutations are epistatic to *ssadh* mutations.

### Peroxides accumulate in *ssadh* mutants but not in *pop2* or *pop2 ssadh* mutants

**Figure 4:**
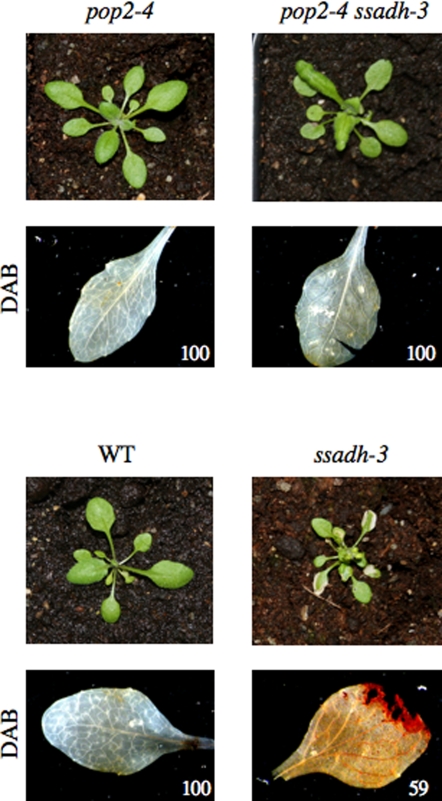
H_2_O_2_ accumulation in *pop2 and ssadh* mutants detected by DAB staining. *In situ* detection of H_2_O_2_ by using 3,3-diaminobenzidine (DAB) staining on *pop2-4*, *ssadh-3*, wild type and *pop2-4 ssadh-3* plants grown at 150 µmol m^−2^ s^−1^ white light (WL) for three weeks (day/night cycle, 16/8 hours; temperature day/night, 20/15°C). Numbers indicate the percentages of leaves showing DAB staining similar to that presented in the pictures (total number of leaves observed was 12 for wild type, 17 for *ssadh-3*, 11 for *pop2-4*, and 15 for *pop2-4 ssadh-3*).

Previously we reported that *ssadh* mutants accumulate high levels of ROIs, which is associated with the severe phenotype of these mutants [26]. To further assess the role of the GABA shunt and its derived metabolites in the *ssadh* phenotype, we tested the accumulation of ROIs in *pop2 ssadh* plants. After three weeks at 150 µmol m^−2^ s^−1^ (day/night cycle, 16/8 hours), leaves of *ssadh*, but not of the wild type, *pop2*, or *pop2 ssadh* mutants, were stained with 3,3-diaminobenzidine (DAB), a substance that detects H_2_O_2_ *in situ* [28] (Figure 4). Thus, only *ssadh* plants accumulate H_2_O_2_ as compared to wild type and *pop2* or *pop2 ssadh*.

**Figure 5:**
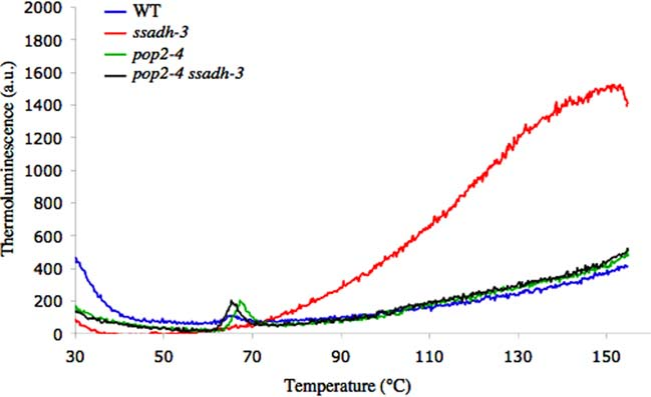
Peroxide accumulation measured by high temperature thermoluminescence emission (HTL) in *pop2* and *ssadh* mutants. High temperature thermoluminescence emission measured in arbitrary units (a.u.) on rosette leaves fixed on aluminum foils using the custom-made apparatus and software described earlier [29]. Wild type (blue), *pop2-4* (green), *ssadh-3* (red) and *pop2-4 ssadh-3* (black) plants were grown *in vitro* for three weeks under standard conditions (day/night cycle, 16/8 hours; light intensity, 100–150 µmol m^−2^ s^−1^; temperature day/night, 20/15 °C). The experiment was repeated 10 times and one representative signal is shown. Thermoluminescence heating rate; 0.1 °C s^−1^.

Peroxide contents were then assessed in the different mutants and the wild type by measuring the thermoluminescence of leaf discs at high temperature. For this, the luminescence of a sample subjected to progressive warming is measured [29], [30]. The chemiluminescence bands observed above 60°C, beyond the temperature at which photosystem II is fully destroyed by heat, are related to the thermolysis of peroxides [31], [32]. High temperature thermoluminescence (HTL) bands are indicators of oxidative stress in the sample, and the intensities of HTL bands observed at 130°C correlates with the lipid peroxide amounts measured in leaves by chemical methods [31]–[33]. Leaves of plants grown under standard conditions were fixed on small aluminum discs, and both, thermoluminescence and fluorescence *F0* were recorded (Materials and Methods). Figure 5 shows representative signals obtained at 0.1°C s^−1^ on leaf samples of wild type, *pop2*, *ssadh* and *pop2 ssadh* plants. The thermoluminescence measured at 135°C is about four times higher in *ssadh* mutants than in wild type or *pop2 or pop2 ssadh* plants, indicating that *ssadh* mutants contain more peroxides (Figure 5). The fluorescence *F0* recorded simultaneously (Figure S2) reaches a peak at about 65°C in the wild type and reflects the heat disruption of photosystem II [34]. The lower heat-induced rise of *F0* fluorescence recorded in *ssadh* mutants as well as the downshift of the *F0/Temperature* curve to lower temperature shows that photosystem II is impaired in *ssadh* plants possibly due to high ROI levels. Thus, neither *pop2* single mutants or *pop2 ssadh* double mutants accumulate peroxides at levels comparable to the ones detected in *ssadh* mutants. More specifically, suppression of the *ssadh* phenotype by mutations in the *POP2*/*GABA-T* gene is associated with elimination of excess ROIs.

### *pop2 ssadh* plants are hypersensitive to SSA and GHB

**Figure 6:**
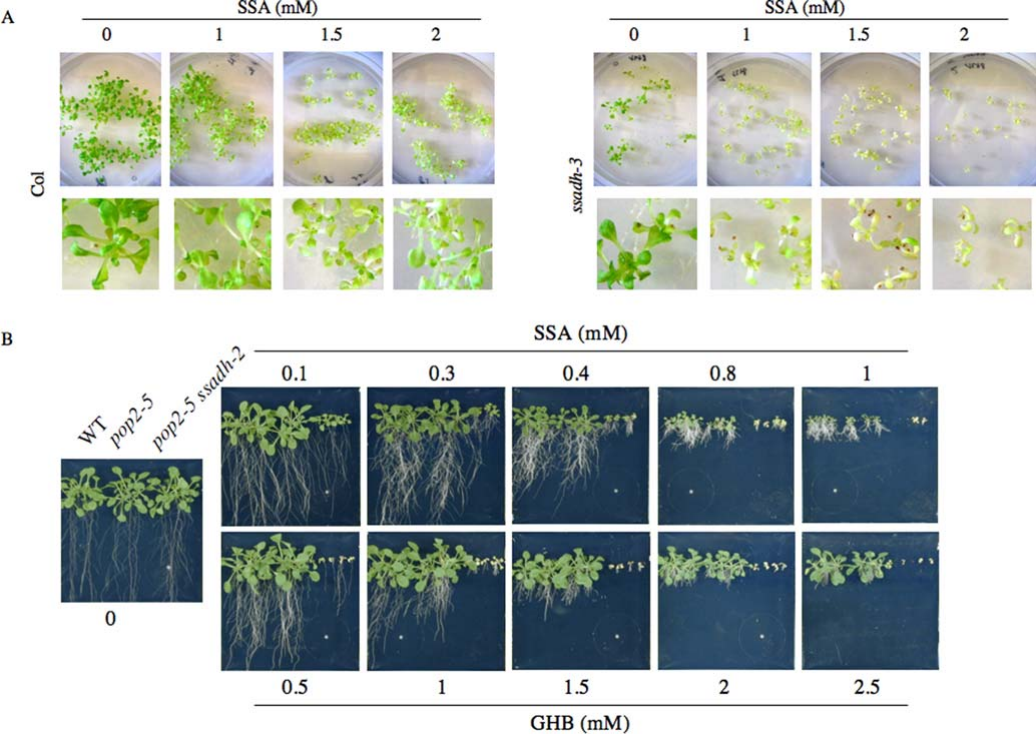
Phenotypes of *ssadh*, *pop2*, *pop2 ssadh* and wild type plants grown on media containing SSA or GHB. (A) Phenotype of the *ssadh-3* mutants and Col-0 wild type plants grown *in vitro* for 14 days under standard conditions (day/night cycle, 16/8 hours; light intensity, 100–150 µmol m^−2^ s^−1^; temperature day/night, 20/15°C) with various amount of succinic semialdehyde (SSA) added to the medium. Photographs of Petri dishes and corresponding magnified pictures are shown. The experiment was repeated three times. (B) Phenotype of Col-0 wild type (WT), *pop2-5* and *pop2-5 ssadh-2* plants grown on ½ MS for 18 days under standard conditions (day/night cycle, 16/8 hours; light intensity, 100–150 µmol m^−2^ s^−1^; temperature day/night, 20/15°C) supplemented with the indicated concentrations of succinic semialdehyde (SSA) or γ-hydroxbutyrate (GHB).

We suspected that the toxic compound responsible for the phenotype of *ssadh* plants and ROIs accumulation might be either SSA or an SSA-derived metabolite such as GHB since mutations in the *GABA-T* gene can rescue the *ssadh* phenotype and only *ssadh* plants accumulate ROIs. To further test this hypothesis, we grew *ssadh-3* and wild type plants *in vitro*, with various amounts of SSA directly added to the culture medium. In contrast to wild type plants, the development of *ssadh-3* seedlings was significantly affected when plants were grown on plates containing 1 mM SSA (Figure 6A). Higher amounts of SSA had an even more drastic effect on the growth of *ssadh-3* (SSA 1.5 and 2 mM; Figure 6A). Thus, our results show that *ssadh* plants are more sensitive to SSA than wild type plants. We then grew wild type, *pop2-5* and *pop2-5 ssadh-2* mutants on plates containing different amounts of either SSA or GHB (Figure 6B). The *ssadh* single mutants were not included in the analysis because the development of the plants is already severely impaired under standard conditions of growth. Whereas the growth of wild type, *pop2*, and *pop2 ssadh* was indistinguishable from one another on agar plates without additional SSA or GHB, growth of the double *pop2 ssadh* mutant was severely affected on low concentrations of SSA or GHB (0.1 mM SSA and 0.5 mM GHB; Figure 6B). With increasing amounts of either compound, growth of the *pop2-5 ssadh-2* mutant was even more affected than that of single *ssadh-3* mutants grown on SSA, most likely due to a more severe phenotype of *ssadh-2* than *ssadh-3* mutants (Figure 6A; [26]). The phenotypes of wild type plants and the *pop2* mutant were similar for all concentrations of SSA and GHB tested (Figure 6B); increased inhibition of plant growth was correlated with increased amounts of SSA or GHB in the medium. Our data show that *pop2 ssadh* plants are more sensitive to GHB or SSA added to the medium than *pop2* single mutants. In animals and possibly in plants, SSA and GHB can be interconverted by the SSA reductase / GHB dehydrogenase (Figure 1A). As both GHB and SSA cannot be properly metabolised in *pop2 ssadh* plants, it is thus likely that one of the two compounds or both are accumulating in a toxic manner in *pop2 ssadh* plants in which levels of SSA or GHB are artificially increased.

**Figure 7:**
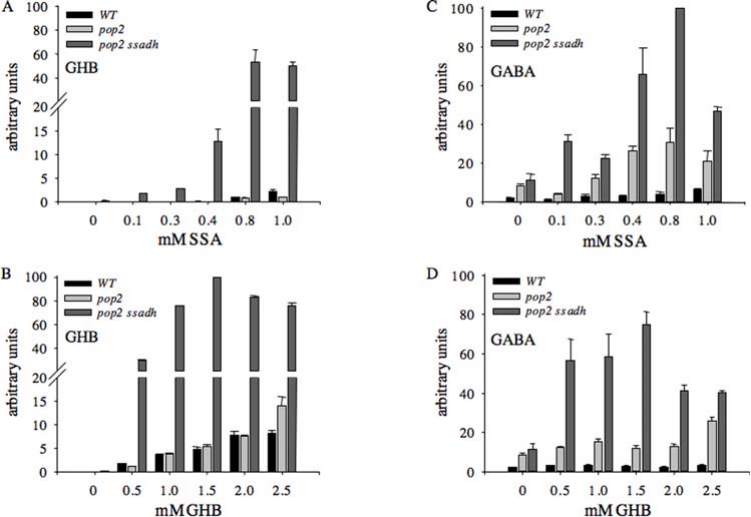
GHB and GABA contents of *pop2*, *pop2 ssadh* and wild type plants grown on either SSA or GHB. (A) and (C) Relative amounts (highest value is 100%) of γ-hydroxbutyrate (A; GHB) and γ-aminobutyrate (C; GABA) in leaves of Col wild type (WT), *pop2-5* and *pop2-5 ssadh-2* mutant plants grown on the indicated concentrations of succinic semialdehyde (SSA). Data (relative arbitrary units normalized to plant weight and the abundance of the internal standard ribitol) were sampled using a GC/MS system, n = 4–8 (WT), n = 2–8 (*pop2*) and n = 2–8 (*pop2 ssadh*)±SE for GHB and n = 4–10 (WT), n = 2–10 (*pop2*) and n = 1–8 (*pop2 ssadh*)±SE for GABA. (B) and (D) Relative amounts (highest value is 100%) of GHB (B) and GABA (D) in leaves of Col wild type (WT), *pop2-5* and *pop2-5 ssadh-2* mutant plants grown on the indicated concentrations of GHB. Data (relative arbitrary units normalized to plant weight and the abundance of the internal standard ribitol) were sampled using a GC/MS system, n = 4–8 (WT), n = 4–8 (*pop2*) and n = 1–8 (*pop2 ssadh*)±SE for GHB and n = 4–10 (WT), n = 4–10 (*pop2*) and n = 2–8 (*pop2 ssadh*)±SE for GABA.

To further assess this hypothesis, GHB contents in leaves of wild type, *pop2-5* and *pop2-5 ssadh-2* plants grown on either SSA or GHB were monitored. To our knowledge, no method is currently available to measure SSA levels in plants. In wild type and *pop2*, GHB is undetectable when grown on ½ MS medium (0 mM SSA or GHB; Figure 7A,B) but accumulates with increasing amounts of GHB added to the medium (Figure 7B). The *pop2 ssadh* mutants were found to accumulate GHB in vast amounts even in response to moderate quantities of SSA (Figure 7A) or GHB (Figure 7B) in the medium. Therefore, *pop2 ssadh* plants accumulate much more GHB in response to either SSA or GHB added to the medium, compared to wild type and *pop2* that both respond in a similar way. Both GHB and SSA added to the medium can therefore be metabolized through the SSADH step in *pop2* or the wild type but not in *pop2 ssadh*. Accumulation of GHB and/or possibly SSA is most likely affecting growth of *pop2 ssadh* on SSA or GHB (Figure 7B). The results indicate that not the lack of succinate or NADH cause the severe *ssadh* phenotype, but rather the accumulation of a toxic compound associated with SSA and/ or GHB metabolism, which is associated with high ROI.

### *pop2 ssadh* plants grown on SSA or GHB accumulate GABA

GABA also accumulates in single *ssadh-2* mutants [14]. To investigate if high amounts of GABA played a role in causing severe phenotypes of *ssadh* plants, we measured GABA levels in wild type, *pop2-5* and *pop2-5 ssadh-2* plants grown on either SSA or GHB. Compared to wild type, leaves of *pop2* and *pop2 ssadh* contained four and five times more GABA, respectively, when grown on medium without SSA (Figure 7C) or GHB (Figure 7D). Thus, disrupting the GABA degradation pathway at the SSADH [14] or the GABA-T step (see also [21]) or both leads to GABA accumulation. The severe phenotypes of *ssadh* plants have been restored in double *pop2 ssadh* mutants to wild type levels, implying that higher GABA contents in these mutants are not impairing growth.

In wild type plants grown on different amounts of SSA or GHB, the GABA content did not differ significantly (with the exception of severely affected plants grown on high SSA), implicating that the GABA pool size of the wild type did not respond to SSA or GHB added to the medium. Interestingly, the *pop2* mutant accumulated higher amounts of GABA than wild type plants, especially in response to high SSA (0.4 mM and higher; Figure 7A) and high GHB (2.5 mM; Figure 7B). The double *pop2 ssadh* mutants accumulated enormous amounts of GABA in leaves in response to even moderate amounts of SSA (0.1 mM; Figure 7C) or GHB (0.5 mM; Figure 7D) in the medium. Hence, GABA accumulates much more in *pop2 ssadh* than in *pop2* in response to either SSA or GHB added to the medium.

## Discussion

The GABA-shunt is composed of three enzymatic steps (Figure 1A), and Arabidopsis knockout mutants were isolated for most corresponding genes (with the exception of *GAD3*, *GAD4* and *GAD5*). The phenotypes of single *pop2* mutants isolated in the present study (*pop2-4* and *pop2-5*) are similar to the ones previously described [21], [25]. In a previous paper, we revealed that a functional SSADH is essential for normal plant growth, and we hypothesized that it might function by suppressing the accumulation of H_2_O_2_ generated under abiotic stresses [26]. One explanation is the ability of the GABA-shunt to supply NADH and/or succinate under conditions that may inhibit the tricarboxylic acid cycle, impair respiration and enhance the accumulation of ROIs. In double *pop2-4 ssadh-3* and *pop2-5 ssadh-2* mutants, the last steps of the GABA-shunt pathway are not functional thus preventing the production of both succinate and NADH by the SSADH enzyme. Still, the double mutant present no signs of oxidative stress and grow like wild type plants in all conditions tested (culture rooms and greenhouses; see also Figure 1, 2 and 4). Indeed, peroxides only accumulate in *ssadh* mutants (Figure 5), consistent with H_2_O_2_ accumulation in these mutants compared to the wild type, *pop2* or *pop2 ssadh* (Figure 4). Thus, the lack of succinate and/or NADH, normally produced by the GABA shunt, is probably not the cause of the accumulation of H_2_O_2_ in *ssadh* plants.

Alternatively, we hypothesized that metabolites derived from the GABA-shunt could accumulate in *ssadh* mutants and be lethal to cells by causing ROIs accumulation [26]. Both GABA and GHB levels are elevated in *ssadh* mutants. Under conditions that favor the production of GABA via the GABA-shunt (e.g. abiotic stresses such as high light, heat or UV), GABA and GHB contents increase in *ssadh-2* [14]. GABA levels also increase as much as four times in leaves of the *pop2-5* mutant (Figure 7C,D) and of other *pop2* alleles [21] as well as in the *pop2 ssadh* double mutant (Figure 7C,D). Thus, impairing the degradation of GABA by inactivating the genes encoding GABA-T and/or SSADH is resulting in GABA accumulation. However, we exclude the possibility that high levels of GABA provoke an oxidative stress in *ssadh* mutants because GABA accumulation in *pop2* or *pop2 ssadh* is not associated with high ROI contents such as in *ssadh* plants.

In plants, POP2 converts GABA to SSA by using pyruvate as amino acid acceptor, producing alanine. However, a second GABA transaminase that remains to be identified, using α-ketoglutarate and producing glutamate was detected in plant extracts [6]. In the present study, we show that GABA accumulates in tremendous amounts in *pop2-5 ssadh-2* compared to wild type plants in response to either SSA or GHB added to the medium (Figure 7). In the wild type, the POP2 enzyme is probably favoring the general equilibrium between GABA and SSA in the direction of GABA degradation, even when SSA is in high excess, like in plants grown on high amounts of SSA. In *pop2*, SSA in excess is metabolized to both GHB and succinate, but in *pop2 ssadh*, the only choice to convert SSA is into GHB since both POP2 and SSADH enzymes are absent. Alternatively, given that an α-ketoglutarate-dependent GABA transaminase exists, SSA could be converted to GABA, too. Another transaminase might exist, which accepts SSA or GABA as side substrates only upon accumulation of either metabolite.

Interrupting the GABA-shunt pathway at the step controlled by the GABA-T enzyme either by inhibiting its activity using Vigabatrin [14], or by disrupting the *GABA-T* gene, prevents the accumulation of ROIs observed in *ssadh* mutants. The metabolite accumulating in *ssadh* plants which is linked to the production of ROIs might thus be produced downstream of the GABA-T transamination step. We have shown in the present study that *ssadh* and *pop2 ssadh* are more sensitive than wild type to SSA added to the medium (Figure 6). Thus, accumulation of SSA itself could be toxic to plants since SSA is a reactive carbonyl and may lead to increased oxidative stress. Accordingly, adding SSA to the culture medium could artificially increase the content of SSA in mutant plants that are already unable to catabolize this toxic metabolite, resulting in even more dramatic effects and increased oxidative stress. Since GHB is derived from SSA, GHB accumulates in the brain of mammalians deficient for the *SSADH* gene [12] as well as in *ssadh* plants [14]. The accumulation of GHB in *ssadh* plants could also be responsible for the oxidative stress generated. Isolating mutants impaired in the conversion of SSA to GHB (Figure 1A) might help to clarify this point. The mechanisms underlying the neurological disorders that predominate in SSADH-deficient patients are poorly understood. Increased GHB and GABA levels result in disturbed neurological functions in mice, but other factors such as high ROI contents are likely to participate in neurodegeneration [35], [36]. Indeed, a recent study shows that GHB induces an oxidative stress in rat cerebral cortex when administrated to high levels [37].

In summary, the results of the current study suggest that the severe phenotype of *ssadh* mutants [26] is not caused by the lack of supply of succinate or NADH to the TCA cycle, but rather due to SSA and/or GHB accumulation that provoke an oxidative stress with elevated levels of ROIs. Whether GHB *per se* is toxic to plants at high concentrations remains to be determined. Still, the similarities of metabolite profiles and ROI accumulation between plant mutants and SSADH-deficient patients raise interesting questions regarding the action of GHB and metabolites of the GABA-shunt in both plants and animals.

## Materials and Methods

### Plant materials and growth conditions

Surface-sterilized seeds were plated on Gamborg B5 medium pH 6.4 (Sigma) containing 1% to 2% sucrose and 0.8% agar (plant cell culture tested, Sigma), incubated at 4°C for 48 hours and grown *in vitro* under the following conditions: day/night cycle of 16/8 hours, light intensity 100–150 µmol m^−2^ s^−1^, temperature day/night was 20/15°C. Seedlings were transferred from plates to soil and grown in controlled-environment chambers. Temperatures and light/dark cycles were the same as for *in vitro* cultures with humidity kept at 65%. Alternatively, ½ MS containing 1% sucrose and 0.8% Gelrite was used.

For SSA and GHB assays, SSA (Sigma; Ref# 14075) and GHB (Sigma; Ref# H3635) were sterile filtered and added to the culture medium, respectively. The pH was adjusted to 5.8 with NaOH.

### Isolation of T-DNA insertion mutants, genotype characterization and mutagenesis

The *ssadh-2* (Col-0) and *ssadh-3* (Col-0) mutants were described previously [26], [38]. The *gaba-t*/*pop2-4* (Col-0) mutant was isolated from the Syngenta T-DNA insertion collection of mutants (line SAIL_1230_C03) and genotyped using forward (5′-GGTTTATGGTGTACTGCC-3′) and reverse (5′-GTACGGTTCCAAAAGGAGTG-3′) oligos specific of the *GABA-T*/*POP2* ORF and Sail T-DNA primers. The *gaba-t*/*pop2-5* (Col-0) mutant was isolated from the GABI_Kat T-DNA insertion collection of mutants (GABI_157D10) and genotyped using forward (5′-CTTTCCCTTTTGGTGTCATTTTTA-3′) and reverse (5′-GGCTAATCTGGTTGAGAACTCC-3′) oligos specific of the *GABA-T*/*POP2* gene and Gabi-Kat T-DNA primers.

For the EMS mutagenesis, seeds were incubated in water containing 0.23% ethylmethane sulfonate (EMS) for 12 hours at room temperature, washed several times with water and dried (Lehle Seeds, Round Rock, USA).

### Analysis of mRNAs expression by RT-PCR

Total RNA extraction was performed with the RNeasy plant kit (Qiagen), followed by a DNAse treatment (Qiagen). RT-PCRs were performed with the OneStep RT-PCR kit (Qiagen) as indicated by the manufacturer. PCR amplification of a 197-bp *GABA-T*/POP2 cDNA specific sequence was performed with a forward (5′-CAGAGTGCTGATTTAGATCCC-3′) and a reverse (5′-GTACGGTTCCAAAAGGAGTG-3′) primer amplifying a region spanning nucleotides 318–514 of the *GABA-T/POP2* cDNA (GenBank accession NM_113117). PCR amplification of a 987-bp *SSADH* cDNA specific sequence was performed with a forward (5′-CTCGTCTCTTCTCAGTGTCGC-3′) and a reverse (5′-CGCTTCAGAAAAGGCCTCAGC-3′) primer amplifying a region spanning nucleotides 133–1119 of the *SSADH* cDNA (GenBank accession AF117335). PCR amplification of the cDNA encoding the elongation factor 1-alpha of Arabidopsis (GenBank accession AY039583) with a forward (5′-GCACTGTCATTGATGCTCC-3′) and a reverse (5′-GTCAAGAGCCTCAAGGAGAG-3′) primer, served as control.

### Detection of ROIs

Thermoluminescence measurements were performed using the custom-made apparatus and software described earlier [29]. Luminescence was detected through the main arm of a multifurcated light guide (14 mm diameter, Walz, Effeltrich, Germany) by a Hamamatsu H5701-50 photomultiplier linked DAQ-Pad-1200 computer interface from National Instruments (Austin, Texas, USA). The constant fluorescence level *F_0_* was simultaneously monitored by pulsing an ultra-weak 480 nm LED once every 5 sampling steps through one arm of the light guide, and *F_0_* was subsequently separated from the luminescence signal by software interpolation [39]. Temperature was controlled by a 4×4 cm Thermatec Peltier element (Melcor, Trenton, NJ, USA) connected to a custom built power supply with a proportional current regulation by computer. To achieve a good thermal contact between small curly leaves of the *ssadh* mutants and the warming plate, leaf samples, the size of which varied from about 1 mm for the *ssadh* mutants to about 1 cm for the wild type, were gently pasted with the upper surface upward on the sticking face of a 25 mm diameter disc punched out of an aluminum foil [29]. The rate of heating during measurement was 0.1°C s^−1^ from 30 to 160°C. Thermoluminescence measurements were repeated several times and representative signals are shown in Figure 5.

H_2_O_2_ was detected *in situ* using 3,3-diaminobenzidine (DAB) as described [28].

### GC-MS measurements

60 to 100 mg plant tissue per sample were harvested in 2 ml tubes and frozen immediately in liquid nitrogen. For metabolite extraction, the tissue was pulverized and mixed with 300 µl cold methanol and 30 µl ribitol (0.3 mg/ml dissolved in methanol; Sigma Ref#A5502). Samples were stored on ice during extraction followed by incubation at 70°C for 15 min. 200 µl chloroform was added, then mixed and incubated for 5 min at 37°C. Finally, 400 µl water (HPLC quality) was added followed by short mixing and 5 min centrifugation at 14.000 rpm. For each aliquot, 160 µl of the upper polar phase were removed and transferred into 1.5 ml glass flask and vacuum dried in a speed vac for about 90 min without heating. Samples could then be stored at −20°C until derivatisation. Derivatisation took place in two steps: first, samples were suspended in 40 µl methoxyamine hydrochloride (20 mg/ml, dissolved in pyridine; Aldrich #226904) and samples were kept at 30°C for 90 min, then 70 µl MSTFA (N-Methyl-N-trimethylsilyltrifluoracetamide, CS-Chromatographie Service GmbH #370520) was added and samples were incubated at 37°C for 30 min. 100 µl of the derivatized extract were then transferred to glass flasks with micro inserts and used for measurement. Measurements were performed using a GS-MS system from Agilent Technologies (injector: model #7683; gas chromatograph: model #6890N; mass selective detector: model #5973, column: model #19091S-433). As carrier gas, He 5.0 was used. 2 µl derivatized sample were injected splitless onto the column. A run consisted of the following steps: first, the oven temperature was kept at 70°C for 5 min, then the temperature was increased with a ramp of 5°C/min up to 280°C, kept there for 7 min and finally, the oven was cooled to 70°C and kept there for 5 min before injecting the next sample. During the whole runtime, the inlet was kept on 250°C, the mass spectrometer at 280°C.

## Supporting Information

Figure S1 Phenotype of double *pop2 ssadh* plants. Phenotype of the *pop2-4 ssadh-3* (A) and *pop2-5 ssadh-2* (B) mutants compared to WT (Col-0) plants and single *pop2* mutants. *ssadh-3* plants are shown as controls (C). Seeds were sown on soil and grown for a total of 45 days in the greenhouse before being photographed.
(6.43 MB PDF)

Figure S2 Fluorescence *F0* measured in wild type and *ssadh-3* mutants. Fluorescence *F0* measured in arbitrary units (a.u.) on leaves fixed on aluminum foils using the laboratory-made apparatus and software described earlier [29]. Wild type (blue dots) and *ssadh-3* (red dots) plants were grown *in vitro* as described in legend of Figure 5. Thermoluminescence (Figure 5) and fluorescence *F0* were measured simultaneously on rosette leaves. Fluorescence *F0* intensities were calibrated to their values at 30°C.
(0.10 MB PDF)
