# Supplementary figures and images for: Mutants of GABA Transaminase (POP2) Suppress the Severe Phenotype of succinic semialdehyde dehydrogenase (ssadh) Mutants in Arabidopsis
Source: PLoS One. 2008 Oct 10;3(10):e3383. doi: 10.1371/journal.pone.0003383 (PMC2557145; doi:10.1371/journal.pone.0003383)

Figure S1

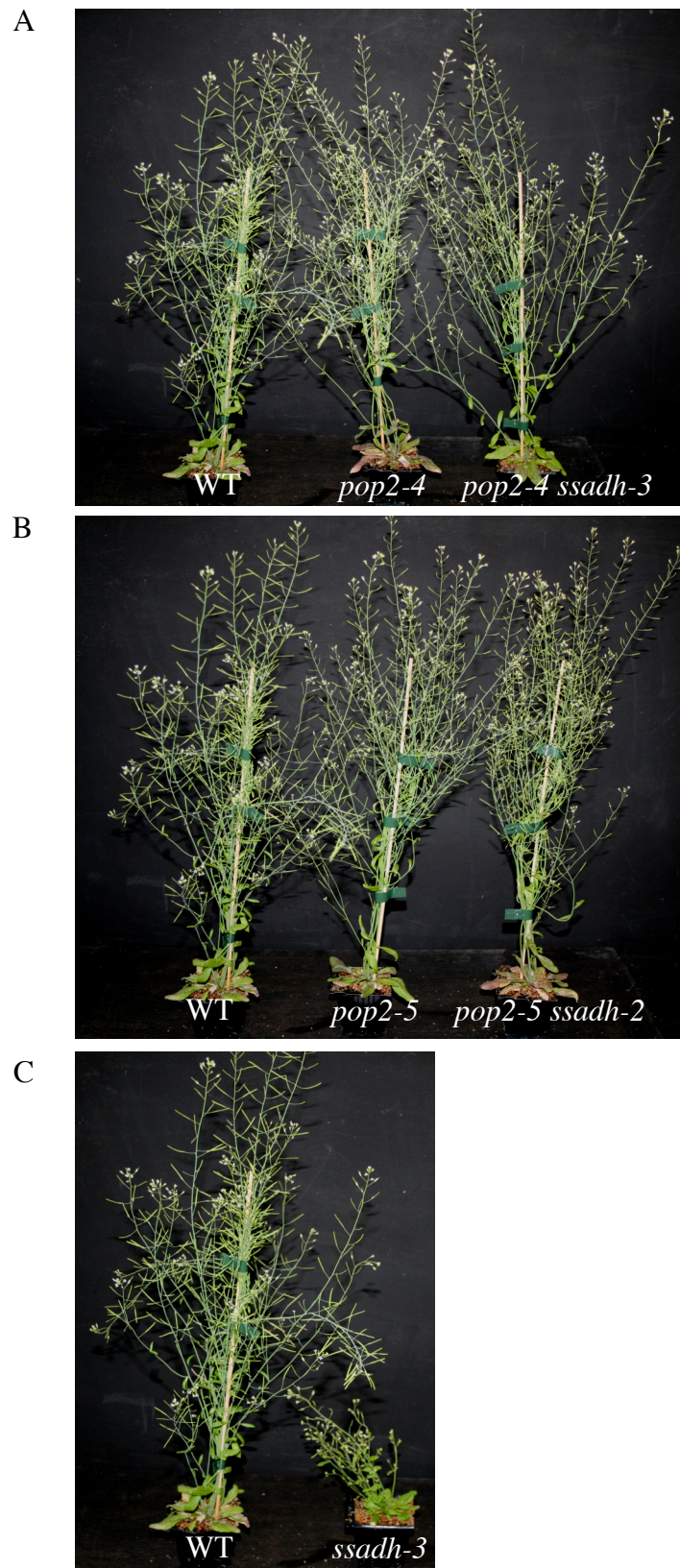

Supplement: Figure S1 — Phenotype of double pop2 ssadh plants. Phenotype of the pop2-4 ssadh-3 (A) and pop2-5 ssadh-2 (B) mutants compared to WT (Col-0) plants and single pop2 mutants. ssadh-3 plants are shown as controls (C). Seeds were sown on soil and grown for a total of 45 days in the greenhouse before being photographed. (6.43 MB PDF) [file pone.0003383.s001.pdf]

Figure S2

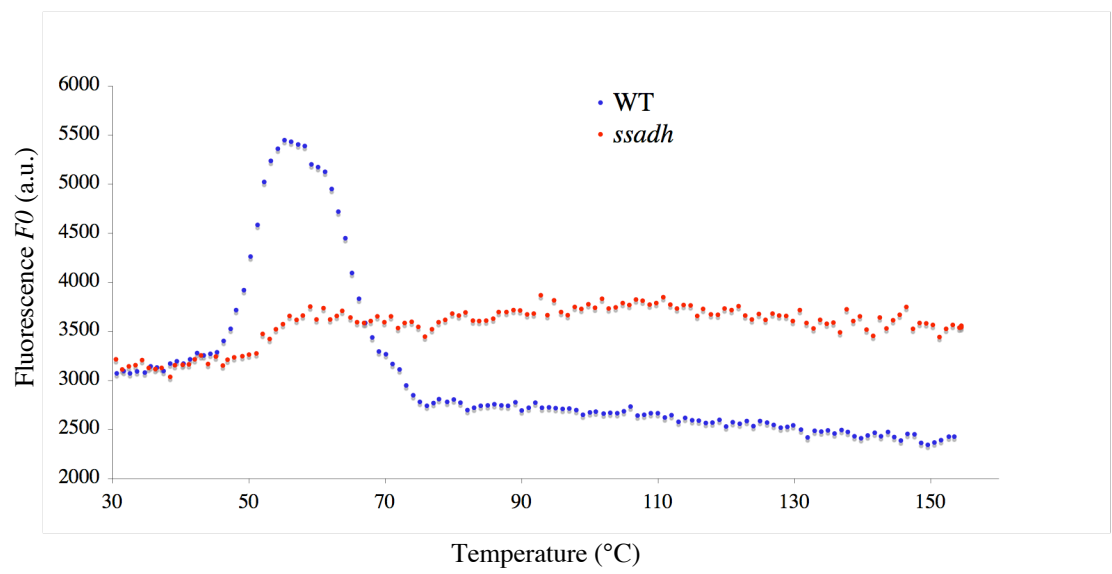

Supplement: Figure S2 — Fluorescence F0 measured in wild type and ssadh-3 mutants. Fluorescence F0 measured in arbitrary units (a.u.) on leaves fixed on aluminum foils using the laboratory-made apparatus and software described earlier [29]. Wild type (blue dots) and ssadh-3 (red dots) plants were grown in vitro as described in legend of Figure 5. Thermoluminescence (Figure 5) and fluorescence F0 were measured simultaneously on rosette leaves. Fluorescence F0 intensities were calibrated to their values at 30°C. (0.10 MB PDF) [file pone.0003383.s002.pdf]
